# Tobacco Harm Reduction with Vaporised Nicotine (THRiVe): A Feasibility Trial of Nicotine Vaping Products for Smoking Cessation Among People Living with HIV

**DOI:** 10.1007/s10461-022-03797-0

**Published:** 2022-07-22

**Authors:** Stephanie Edwards, Cheneal Puljević, Judith A. Dean, Charles Gilks, Mark A. Boyd, Peter Baker, Peter Watts, Chris Howard, Coral E. Gartner

**Affiliations:** 1 School of Public Health, The University of Queensland, Brisbane, Australia; 2 Centre for Health Services Research, The University of Queensland, Brisbane, Australia; 3 Faculty of Health & Medical Sciences, University of Adelaide, Adelaide, Australia; 4 Northern Adelaide Local Health Network (NALHN), Adelaide, Australia; 5 Kirby Institute, University of New South Wales, Sydney, Australia; 6 Queensland Positive People, Brisbane, Australia

**Keywords:** Tobacco, HIV, Vaping, Smoking cessation

## Abstract

People living with HIV (PLHIV) have high rates of tobacco smoking. Nicotine vaping products (NVPs) may promote tobacco smoking cessation and/or harm reduction. This study aimed to trial the feasibility of NVPs for promoting tobacco smoking cessation among PLHIV. The Tobacco Harm Reduction with Vaporised Nicotine (THRiVe) study was a mixed-methods trial among 29 PLHIV who used tobacco daily. Participants trialled a 12-week intervention of NVPs. This study reports descriptive analyses of quantitative data on tobacco abstinence and associated adverse events. Short-term abstinence (7-day point prevalence; i.e., no tobacco use for 7 days) was achieved by 35% of participants at Week 12 and 31% reported short-term abstinence at Week 24. Sustained medium-term abstinence (8 weeks’ abstinence) was achieved by 15% of participants at Week 12 and 31% at Week 24. Most adverse events were mild. NVPs may represent a feasible and potentially effective short-to-medium term tobacco smoking cessation aid and/or harm reduction strategy among PLHIV.

**Supplementary Information:**

The online version contains supplementary material available at 10.1007/s10461-022-03797-0.

## Introduction

Internationally, people living with human immunodeficiency virus (PLHIV) are two to three times more likely to smoke tobacco than the general population [1–3]. As a result, tobacco smoking is a significant risk factor for morbidity and premature mortality among PLHIV [4–8]. For example, a study of 17,995 PLHIV in Europe and North America estimated that PLHIV lose more years of life from tobacco smoking than from HIV itself [6]. Reasons for the high rates of tobacco use among PLHIV are complex and overlapping [9], and include socioeconomic determinants such as unemployment, lower education or income levels [10–12], experiences of mental illness and/or substance use disorders [13–15], and/or using tobacco to manage stress associated with living with HIV [16, 17].

Despite the negative health impacts of tobacco smoking for PLHIV [4, 6–8], a recent systematic review and meta-analysis found that HIV diagnosis or treatment initiation did not typically result in quitting smoking [9]. Evidence suggests that many PLHIV have attempted to quit tobacco smoking; however, most attempts are unsuccessful [18–20]. These low quit rates may be influenced by a low level of interest in using tobacco smoking cessation prescription medications, nicotine replacement therapy (NRT) or behavioural therapy for quitting tobacco smoking [16, 21–23]. Furthermore, a Cochrane review of the effectiveness of behavioural and pharmacological tobacco cessation interventions among PLHIV suggests that many of these interventions are effective at promoting short-term abstinence only, with relapse to tobacco smoking common after the intervention is stopped [24].

In the general population, there is evidence from observational studies and randomised controlled trials that nicotine vaping products (NVPs; e.g., e-cigarettes) can increase the likelihood of successful tobacco smoking cessation [25–27]. A recent Cochrane review [28] incorporating 61 studies concluded that there is moderate‐certainty evidence that NVPs increase tobacco smoking quit rates compared to vaping products without nicotine, behavioural support or NRT. The review did not identify any evidence of harms associated with NVP use, but the authors acknowledged the limited duration of follow-up in these studies [28]. In addition to their potential as an effective tobacco smoking cessation aid, NVPs are often considered a substitute for tobacco smoking [29–31]. While some studies have identified potentially harmful constituents in NVPs [32], the National Academies of Science, Engineering and Medicine Consensus Report concluded that there “is conclusive evidence that completely substituting e-cigarettes for combustible tobacco cigarettes reduces users’ exposure to numerous toxicants and carcinogens present in combustible tobacco cigarettes” and that while the absolute risks associated with NVP use have not been determined, NVPs are “likely to be far less harmful than combustible tobacco cigarettes”. As a result, NVPs may be a unique harm reduction option that could be used to prevent subsequent tobacco smoking relapse for those who may benefit from a longer-term substitute [29].

NVPs have been suggested as a potentially effective tobacco smoking cessation strategy for PLHIV [33]. A study of 25 PLHIV in the USA found that participants were willing to try NVPs, but were uncertain of their safety and efficacy [34]. Furthermore, a focus group study involving 54 PLHIV in Australia found that NVPs were the tobacco cessation product that participants were most interested in using for a quit attempt, and were perceived as more suitable than NRT for long-term use [35]. To our knowledge, only one short duration (12 weeks) pilot trial of 19 USA-based PLHIV has tested the acceptability and feasibility of NVPs in PLHIV [36]. This study reported significant reductions in cigarettes smoked per day, cigarette dependence scores and carbon monoxide levels, and significantly improved motivation to quit tobacco smoking after eight weeks of NVP use. Our study, the ‘Tobacco Harm Reduction with Vaporised Nicotine’ (THRiVe) clinical trial, adds to this limited body of evidence. In this study, we examined the feasibility of NVPs as a tobacco smoking cessation and/or harm reduction intervention among a sample of PLHIV who smoke tobacco by assessing NVPs’ acceptability, effectiveness and safety.

## Methods

The THRiVe study used a mixed-methods design to assess the safety, effectiveness and acceptability of using a NVP for 12 weeks as a tobacco smoking cessation and/or harm reduction intervention among a sample of PLHIV who smoke tobacco. Quantitative survey data were collected at baseline and Week 4, 8, 12 and 24, and qualitative data at baseline and Week 12, to examine participants’ acceptability and use of the study intervention, attitudes to tobacco smoking, and the impact of NVP use on tobacco smoking behaviour and adverse events. This manuscript presents analyses of quantitative data only. A detailed study protocol has been published [37]. The study was approved by Metro South Human Research Ethics Committee (registration number EC00167). and ratified by The University of Queensland’s Human Research Ethics Committee. The study’s Universal Trial Number (UTN) was U1111-1179-4374 and the study was registered with the Australian New Zealand Clinical Trials Registry (ANZCTR number: ACTRN12616001641482).

### Participants

We recruited PLHIV who smoked tobacco daily from community organisations and health services in Brisbane, Australia, in 2017. Eligibility criteria included: a diagnosis of HIV; aged ≥ 18 years; smoked ≥ 5 cigarettes per day at time of trial enrolment; had been smoking tobacco daily for at least 12 months; and was willing to attempt to quit smoking tobacco after study enrolment. Exclusion criteria included: participating in another tobacco smoking cessation program; pregnant or planning to become pregnant during trial participation period; breastfeeding or planning to be during trial participation period; experienced chest pain or a cardiovascular event or procedure (e.g., heart attack, stroke, insertion of stent, bypass surgery) in the last month; or being treated with oxygen therapy.

### Recruitment and Enrolment

The study was advertised through a community support organisation for PLHIV, three general practitioner clinics and two public sexual health and HIV clinics using posters, advertising cards and word of mouth. Potential participants either contacted researchers directly or were referred to researchers by the services advertising the study. Potential participants completed a short eligibility screening survey with a researcher by telephone, and were provided with a detailed explanation of the study information and consent forms. Alternatively, potential participants completed eligibility screening online and were subsequently contacted by a researcher. Eligible and willing participants who provided informed consent were enrolled in the study.

### Intervention Description

At baseline, each participant was provided with two NVP kits (one Innokin Endura T18® kit and one Innokin Endura T22® kit). Each kit comprised one vaporiser, a tank with a coil, four additional coils, one wall charger, a charging cable, ten 10 mL bottles of unflavoured Nicophar® nicotine e-liquid (12 mg/mL of nicotine in glycerol and purified water), and printed instructions and information on safe use, quitting smoking and NVP use. Additional e-liquid and coils were supplied at Week 4 and 8 as required.. The NVP devices were selected due to their ease of use, manufacturing quality assurance (e.g., Quality Control Certifications provided by the manufacturer) and safety features (electrical safety cut-offs, leak-proof design). Offering two different models of the device catered to individual user preferences and tobacco smoking patterns and allowed participants to choose the most practical device for their tobacco smoking pattern and situation. For example, the Endura T22 has both a larger tank (4 mL) and longer battery life (2000 mAh) than the Endura T18. As a result, the T22 did not require refilling and recharging as regularly as the T18 and therefore may have been preferred by those who smoked more heavily. However, the T22 is a larger and heavier device.

Participants were required to set a ‘start vaping date’ within one week of enrolment and were contacted on or soon after this date to address any queries. The 12 weeks immediately following the ‘start vaping date’ was considered the ‘treatment period’. During the treatment period, participants were asked to make a quit attempt and replace as many tobacco cigarettes as possible with NVP use. Participants were able to engage in ‘dual use’ where they used both cigarettes and NVPs, however researchers made it clear to participants that the aim was to substitute all cigarettes with NVP use. Participants were required to return their NVP kits to the study investigators at the end of Week 12. Participants were reimbursed by electronic funds transfer to cover transport costs associated with attending face-to-face interviews and for the time involved in completing data collection throughout the study (baseline survey and interview, $50; Week 4 survey, $20; Week 8 survey, $20; Week 12 survey and interview, $50; Week 24 survey, $20).

### Data Collection and Outcome Measures

Data were collected either in person or by telephone-administered surveys at baseline and at Weeks 4, 8, 12 and 24. At baseline, we used quantitative surveys to collect data on participant demographic characteristics, number of cigarettes per day (CPD), history of tobacco smoking and quitting (including NRT use), knowledge of health effects of tobacco smoking and nicotine, motivating reasons to quit, quitting self-efficacy, level of nicotine dependency using the Fagerström Test for Nicotine Dependence (FTND) [38], level of behavioural dependence on tobacco smoking using the Glover-Nilsson Smoking Behavioural Questionnaire (GN-SBQ) [39], and familiarity with, past use of, and attitudes towards NVPs.

Follow-up quantitative surveys administered at Weeks 4, 8, 12 and 24 measured attitudes towards and use of the intervention, CPD, quit attempts, adverse events, and abstinence measures. Abstinence measures included participants’ self-report of smoking tobacco daily, non-daily or not at all (self-reported by participant, not considering the number of days since smoking tobacco). Short-term abstinence was assessed and defined as 7-day point prevalence (7-day PP; did not smoke any tobacco in the previous seven days) and medium-term abstinence (defined as self-reported abstinence from tobacco smoking for at least eight weeks; measured at Weeks 8, 12 and 24 only). Short-term and medium-term abstinence were not mutually exclusive categories. The Week 12 survey also re-assessed knowledge of health effects of tobacco smoking and nicotine, quitting self-efficacy, and dependency measures (FTND [38]; GN-SBQ [39]) measured at baseline, and familiarity with, use of and attitudes toward NVPs (including acceptability in terms of assisting with quitting and reducing cravings; ease of use; and comparisons to varenicline, bupropion and NRT). In this paper, we report participants’ demographic characteristics at baseline, the impact of the 12-week intervention on tobacco smoking behaviour (including on abstinence, CPD, quit attempts, quitting self-efficacy and nicotine dependency) and adverse events. All data were collected by the primary author (SE).

### Adverse Events

Adverse events were monitored at each contact with participants. Participants were also advised to contact the researchers when experiencing any health issues. All adverse events were evaluated and assigned an intensity level (mild, moderate, severe), causality rating (five-point scale of ‘not related’ to ‘definitely related’), expectedness rating (expected or unexpected), outcome (recovered, recovered with sequalae, ongoing, death or other) and any treatment documented. Each event was then categorised as an adverse event (defined as any unfavourable and unintended symptom or event), a serious adverse event (symptoms or events that were life threatening or resulted in death, disability or hospitalisation) or a suspected unexpected serious adverse reaction (adverse events causally related to the NVPs that were both serious and unexpected).

### Data Management and Analysis

All quantitative data were entered and stored in REDCap [40], a secure web-based data storage and management application, hosted at The University of Queensland. Quantitative data were then analysed in SPSS [41]. Descriptive statistics (counts, percentages and means) were performed to illustrate direction for all quantitative results. No further statistical tests were conducted as this was a feasibility study with a modest sample size. Baseline data are reported for all participants. Follow up data are reported for participants who were not lost to follow-up by the end of the treatment period (i.e., with available data for both baseline and week 12), to assist with the interpretation of results.

## Results

### Participant Demographics and Tobacco Smoking Practices at Baseline

We recruited 29 PLHIV who smoked tobacco (28 male, 1 non-binary) with an average age of 42 years (Standard Deviation (SD) 8). About half of the participants (52%) had completed post-high school education (e.g., tertiary diploma, trade certificate or university degree; Table 1). The average age of initiating daily tobacco smoking was 16 years (SD 5) and participants had smoked tobacco for an average of 27 years (SD 10). Participants most commonly reported a low to moderate nicotine dependency (FTND; 38% low to moderate) and two thirds reported moderate behavioural dependency (GN-SQB; 66%) (Table 1).

**Table 1:** Participant demographic characteristics, measures of nicotine dependency, past quit attempts and attitudes to quitting at baseline (n = 29)

Characteristic/measure	*n*	%
Highest educational qualification		
Up to year 10	10	34.5
Year 12	4	13.8
Tertiary diploma/ trade certificate/ TAFE^a^	11	37.9
University degree	4	13.8
Ever used nicotine replacement therapy	24	82.8
Nicotine dependency (FTND)^b^		
Low (1–2)	4	13.8
Low-moderate (3–4)	11	37.9
Moderate (5–7)	10	34.5
High (8 +)	4	13.8
Behavioural dependency (GN-SBQ)^c^		
Mild (< 12)	3	10.3
Moderate (12–22)	19	65.5
Strong (23–33)	7	24.1
Main reason to quit		
Health concerns	26	89.7
Financial impact	2	6.9
Social stigma	1	3.4
Made a previous quit attempt	27	93.1
Quitting self-efficacy for quitting unaided/cold turkey		
Very easy	0	0.0
Somewhat easy	1	3.4
Not easy but not too difficult	5	17.2
Somewhat difficult	3	10.3
Very difficult	16	55.2
Impossible	4	13.8
Quitting self-efficacy for quitting using pharmacotherapy		
Very easy	1	3.4
Somewhat easy	4	13.8
Not easy but not too difficult	7	24.1
Somewhat difficult	8	27.6
Very difficult	3	10.3
Impossible	1	3.4
Don’t know	5	17.2
Use of nicotine products in a previous quit attempt^d^		
Nicotine patch	14	48.3
Nicotine gum	10	34.5
Nicotine lozenges	3	10.3
Nicorette inhalator	2	6.9
Nicotine mouth spray	3	10.3
Nicotine dissolvable oral strips	2	6.9
Use of other quit methods (not involving nicotine replacement) in a previous quit attempt		
Cold turkey	23	79.3
Cutting down the number of cigarettes smoked per day, without using any tobacco smoking cessation aids	22	75.9
Varenicline/Champix	20	69.0
Bupropion/Zyban	6	20.7
Quitline counselling	4	13.8
A tobacco smoking cessation group course	2	6.9
Online tobacco smoking cessation program or information	0	0.0
Tobacco smoking cessation text messaging program or app	6	20.7
Future tobacco smoking cessation preference		
Nicotine replacement therapy	6	20.7
Cold turkey	4	13.8
Prescription medicine	4	13.8
Support groups or services (e.g., Quitline counselling, tobacco smoking cessation group course, online program, or phone app)	1	3.4
Nicotine vaporiser	14	48.3

^a^TAFE (Technical and Further Education)

^b^FTND (Fagerström Test for Nicotine Dependence) [38]

^c^GN-SBQ (Glover-Nilsson Smoking Behavioural Questionnaire) [39]

^d^n = 8 participants had also used nicotine replacement therapy during non-quit attempt periods. Reasons for use included being unable to smoke (such as when travelling or in hospital) or participation in research studies

Health concerns were the primary reason for wanting to quit tobacco smoking (90%) at baseline. Most participants (93%) had made at least one quit attempt in the past, with unaided quitting (‘cold turkey’) being the most common previously used method to quit tobacco smoking (79%). Almost three quarters of participants (76%) had previously tried to cut down the number of cigarettes they smoked per day (without a tobacco smoking cessation aid), while 69% had used varenicline to quit tobacco smoking. Nicotine patches were the most commonly used NRT product, used by 48% of participants, while 35% had used nicotine gum. Despite the high rates of self-reported quitting without a quit aid, the majority of participants believed unaided quitting was ‘very difficult’ (55%) or impossible (14%). More than half of the sample believed that quitting tobacco smoking using pharmacotherapy (i.e., prescription tobacco smoking cessation medications or NRT) would be either somewhat difficult (28%) or not easy but not too difficult (24%).

### Tobacco Smoking Abstinence, Dependency and Quit Attempts

**Fig. 1:**
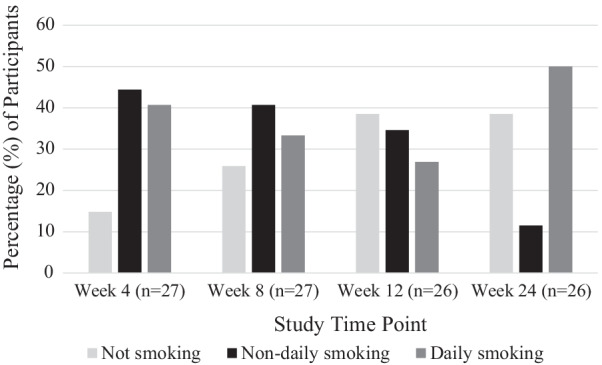
Participants’ tobacco smoking status across study time-points

Three participants were lost to follow-up, two after enrolment and one after the Week 8 survey. As a result, 27 participants were included in Week 4 and 8 data analyses, and 26 participants in analyses of Week 12 and 24 data. Table 2 and Fig. 1 show that the number of participants who achieved tobacco smoking abstinence increased throughout the treatment period. At the end of treatment (Week 12), 35% of participants had achieved short-term abstinence (7-day PP) and 15% had achieved medium-term abstinence. At the 6-month follow-up (Week 24), 31% of participants had achieved medium-term abstinence. The majority of participants (60% or more) tried to completely stop smoking tobacco at least once during each four-week block of the 12-week treatment period.

**Table 2:** Cigarette abstinence measures and quit attempts

	N (%)			
	Week 4 (n = 27)	Week 8 (n = 27)	Week 12 (n = 26)	Week 24 (n = 26)
Short-term abstinence (7-day PP)	3 (11.1)	6 (22.2)	9 (34.6)	8 (30.8)
Medium-term abstinence		1 (3.7)	4 (15.4)	8 (30.8)
No longer smoking tobacco	4 (14.8)	7 (25.9)	10 (38.5)	10 (38.5)
Average number of days of non-smoking (SD)	14 (11.5)	34.7(21.4)	41.9 (30.8)	103.9 (58.4)
No longer vaping	–	–	2 (7.7)	2 (7.7)
Non-daily tobacco smoking	12 (44.4)	11 (40.7)	9 (34.6)	3 (11.5)
Average number of days of non-daily smoking (SD)	15.8 (8.1)	33.9 (17.9)	51.1 (31.24)	99.7 (68.7)
Daily tobacco smoking	11 (40.7)	9 (33.3)	7 (26.9)	13 (50.0)
Attempts to stop smoking tobacco completely (of those still smoking at follow-up point)				
Did not attempt to stop	7 (30.4)	8 (40.0)	4 (25.0)	13 (81.3)
Tried to stop, but lasted less than 1 day	3 (13.0)	4 (20.0)	1 (6.3)	1 (6.3)
Stopped smoking tobacco for 1 day or longer	13 (56.5)	8 (40.0)	11 (68.8)	2 (12.5)

Short-term abstinence: 7-day point prevalence (did not smoke any tobacco in the previous seven days); Medium-term abstinence: did not smoke any tobacco for at least eight weeks; *SD* standard deviation; *PP* point prevalence

Table 2 shows that there was an initial rise in non-daily tobacco smoking at Week 4, which then declined at Weeks 8 and 12. This occurred alongside a steady increase in the number of participants who reported that they were no longer smoking tobacco over the 12-week treatment period. At Week 12, when the NVPs were returned to investigators, there was a sharp rise in daily smoking; 27% of participants reported smoking tobacco daily at week 12, which increased to 50% at Week 24. Most participants who reported non-daily tobacco smoking at Week 12 had returned to daily tobacco smoking by the Week 24 follow-up; non-daily tobacco smoking rates decreased from 35% at Week 12 to 12% by Week 24.

Of the nine participants (35%) smoking on a non-daily basis at the end of the treatment period (Week 12), two (22%) maintained non-daily smoking and three (33%) had progressed to quitting at Week 24 (see Table 3). Seven (70%) of the participants who had quit at Week 12 remained quit at Week 24. Nine participants (35%) reported using an NVP between the end of treatment and the Week 24 follow-up; three of these progressed to less frequent tobacco smoking during this period (i.e., went from smoking tobacco daily to non-daily, or from non-daily tobacco smoking to quitting). An additional three participants maintained their tobacco smoking abstinence or non-daily tobacco smoking status.

**Table 3:** Participants’ change in tobacco smoking status after the treatment period

Week 12			Week 24			Number of participants who used an NVP between Week 12 and 24
Tobacco Smoking Status	*n*	(%)	Tobacco Smoking Status	*n*	(%)	
Quit	10	(38)	Quit	7	(70)	2
			Non-daily	0	(0)	0
			Daily	3	(30)	1
Non-daily	9	(35)	Quit	3	(33)	2
			Non-daily	2	(22)	1
			Daily	4	(44)	1
Daily	7	(27)	Quit	0	(0)	0
			Non-daily	1	(14)	1
			Daily	6	(86)	1

Supplementary Figure 1 illustrates the average CPD across the study of participants who continued to smoke tobacco. A dramatic decline in the average number of cigarettes smoked per day occurred between Baseline (18 CPD) and Week 4 follow-up (6 CPD), where it remained at Week 8. CPD rose at Week 12 (8 CPD) and at Week 24 (14 CPD) after the NVPs were returned at Week 12.

We also noted a reduction in average nicotine dependency scores across the study’s duration. Participants still smoking tobacco at Week 12 had an average FTND score of four (low-moderate dependency) compared to five (moderate dependency) at baseline. A similar trend occurred with behavioural dependence, although a greater reduction was evident; the average GN-SBQ was 12 (mild/moderate dependency) at Week 12 compared to 18 (moderate dependency) at Baseline.

### Adverse Events

A total of 41 adverse events were recorded during the duration of the study (see Supplementary Table 1). No serious or suspected unexpected serious adverse events were recorded. Of the 41 events, 28 events experienced by 16 participants were considered to be possibly, probably or definitely related to the study treatment. Of the 28 adverse events, 22 (79%) occurred within the first four weeks of the treatment period, and 22 (79%) were rated as mild in intensity. The most commonly-reported adverse events were throat irritation (n = 8 occurrences; 29%), experienced by seven (27%) participants, and headaches (n = 7 occurrences; 25%), experienced by four (15%) participants.

## Discussion

This study found that 35% of a sample of PLHIV had achieved short-term tobacco abstinence (self-reported 7-day PP) and 15% had achieved medium-term abstinence (at least eight weeks of self-reported abstinence) after 12 weeks of NVP use. Furthermore, 31% reported medium-term abstinence at Week 24. These findings suggest that NVPs may be a beneficial tobacco harm reduction approach for PLHIV, and that the effectiveness of NVPs for promoting tobacco smoking cessation among PLHIV should be explored in a powered, randomised clinical trial. This result is supported by a US-based pilot study that found that participants reduced their CPD, and some achieved tobacco smoking cessation in both the short and medium-term, after eight weeks of NVP use [36].

These results are promising when compared to findings of a systematic review showing that traditional behavioural and pharmacological cessation interventions among PLHIV resulted in pooled short-term and long-term tobacco smoking abstinence rates of 13% and 8% respectively [24]. Our findings also align with previous research of NVPs in the general population. For example, a study of 5,124 US-based adults who smoked daily found that those who initiated daily NVP use were approximately eight times more likely to quit tobacco smoking compared to non-users [42]. While further research on NVPs’ effectiveness for tobacco smoking cessation and NVP-related harms is still needed [43], our findings demonstrate that NVPs have the potential to be a novel, feasible and effective tobacco smoking cessation treatment for PLHIV.

We also found that participants who did not quit smoking tobacco reduced the average number of cigarettes that they smoked during the study’s treatment period. Dual cigarette NVP use has been associated with reductions in the number of cigarettes smoked in previous research [42, 44]. Reduced tobacco smoking can act as an important first step to quitting [45], particularly among people who perceive themselves as unable to quit [46]. Reduced tobacco smoking also increases the likelihood of making a quit attempt and achieving abstinence, particularly if it occurs in combination with the use of cleaner sources of nicotine [45, 47].

Results illustrated a sharp rise in daily tobacco smoking from week 12 to Week 24. This is most likely due to the return of the NVPs to the trial investigators at Week 12. Longer access (greater than 12 weeks) to NVPs may increase rates of sustained tobacco abstinence [48]. At the Week 24 follow-up, just over one-third of our participants reported using an NVP after the treatment period, and the majority of these participants either maintained abstinence or non-daily tobacco smoking from Week 12, or progressed to less frequent tobacco smoking (i.e., changed from smoking tobacco daily to non-daily, or from smoking tobacco non-daily to quitting). These findings suggest that NVP use, especially over a longer period of time, could help PLHIV to quit smoking tobacco or reduce the frequency of tobacco smoking, and that NVPs are considered acceptable by this population as a quit aid [27]. Further long-term studies of NVP use among PLHIV are needed to support this finding.

### Strengths and Limitations

A major strength of this study is its novelty. To our knowledge, only one other published study has trialled NVPs among PLHIV, but did not report abstinence measures and included only 19 participants [36]. A second strength of the study was the pragmatic approach used, with participants instructed to use the NVPs ad libitum*.* This replicated the real-world use of these products, allowing participants to tailor their use to their individual needs and tobacco smoking behaviour. It also avoided the need to enforce strict adherence criteria [49] and may be one reason for the study’s high participant retention rate, with almost 90% of participants retained for the full six months. Low participant retention rates are a common limitation observed in other tobacco smoking cessation intervention studies among PLHIV [50–53].

Our findings are subject to some limitations. First, due to the lack of a control group and modest sample size, it is difficult to determine the extent to which the results are directly due to the intervention itself. Further controlled studies with larger samples are needed. However, given the novelty of this study and the challenges of identifying effective tobacco smoking cessation interventions with PLHIV, this feasibility study was considered an important first step. Second, our sample was almost entirely male, but this largely reflects the demographics of the population of Australians living with HIV [54]. A third limitation of this study is that the nature of our recruitment strategy resulted in participants who were interested in quitting tobacco smoking and using NVPs, limiting the generalisability of these results to PLHIV who do not wish to quit tobacco smoking or are not interested in using NVPs. However, the results of Cioe et al. [36] suggest that PLHIV who are not ready to quit smoking tobacco in the next month may be willing to use NVPs as an alternative to smoking tobacco. It is also unknown whether there are any health benefits for individuals who achieve temporary short-term tobacco smoking abstinence only. However, temporary abstinence may increase confidence in one’s ability to quit tobacco smoking permanently on future attempts and evidence suggests that “unsuccessful” quit attempts are part of the natural process of quitting tobacco smoking permanently for most people and can provide valuable learnings that increase their ability to achieve permanent abstinence [55]. Furthermore, although our data were collected in 2017, our findings remain relevant as the NVPs provided to participants are still widely available for purchase, and we are only aware of one other published pilot trial of NVPs among PLHIV, conducted in the USA [36]. Finally, limited study resources prevented self-reported abstinence to be biochemically confirmed. Self-reported abstinence may over-state abstinence rates and it would be important in a future, larger randomised control trial to include biochemical confirmation.

## Conclusion

This study found that the provision of NVPs for 12 weeks was associated with 7-day point prevalence tobacco smoking abstinence among 35% of a sample of PLHIV who smoked tobacco daily. These findings suggest that NVPs represent a potentially feasible and effective short-to-medium term tobacco smoking cessation aid and/or tobacco harm reduction strategy among PLHIV. Future research should investigate the effectiveness of NVPs compared to traditional tobacco smoking cessation interventions among PLHIV using more robust, longer duration trial designs.

## Supplementary Information

Below is the link to the electronic supplementary material.

Supplementary file1 (DOCX 144 KB)

Supplementary file2 (DOCX 16 KB)

## Data Availability

The data underlying this article cannot be shared publicly due to protecting the privacy of individuals that participated in the study. The data will be shared on reasonable request to the corresponding author where human research ethics approval allows the de-identified data to be shared.
