# Supplementary material for: Tobacco Harm Reduction with Vaporised Nicotine (THRiVe): A Feasibility Trial of Nicotine Vaping Products for Smoking Cessation Among People Living with HIV
Source: AIDS Behav. 2022 Jul 22;27(2):618–27. doi: 10.1007/s10461-022-03797-0 (PMC9908735; doi:10.1007/s10461-022-03797-0)
Supplement: Supplementary file 1 — Supplementary file1 (DOCX 144 KB) [file 10461_2022_3797_MOESM1_ESM.docx]

**Supplementary Figure 1. Average number of cigarettes per day among participants still smoking**
