# Supplementary material for: Tobacco Harm Reduction with Vaporised Nicotine (THRiVe): A Feasibility Trial of Nicotine Vaping Products for Smoking Cessation Among People Living with HIV
Source: AIDS Behav. 2022 Jul 22;27(2):618–27. doi: 10.1007/s10461-022-03797-0 (PMC9908735; doi:10.1007/s10461-022-03797-0)
Supplement: Supplementary file 2 — Supplementary file2 (DOCX 16 KB) [file 10461_2022_3797_MOESM2_ESM.docx]

**Supplementary Table I. Adverse Events Considered Related to the Study Treatment**

|  | Events reported | Number of participants who experienced the AE |
| --- | --- | --- |
| Adverse Event | *n* (%) | *n* (%) |
| Throat irritation | 8 (29) | 7 (27) |
| Headache | 7 (25) | 4 (15) |
| Cough/chest irritation | 5 (18) | 4 (15) |
| Nausea | 4 (14) | 3 (12) |
| Breathing difficulty | 1 (4) | 1 (4) |
| Gastroesophageal reflux issues and oesophagitis | 1 (4) | 1 (4) |
| Dizziness | 1 (4) | 1 (4) |
| Heart palpitations | 1 (4) | 1 (4) |
